# The Combination of Arginine Deprivation and 5-Fluorouracil Improves Therapeutic Efficacy in Argininosuccinate Synthetase Negative Hepatocellular Carcinoma

**DOI:** 10.3390/ijms18061175

**Published:** 2017-06-01

**Authors:** Angkana Thongkum, Chunjing Wu, Ying-Ying Li, Medhi Wangpaichitr, Panida Navasumrit, Varabhorn Parnlob, Thaniya Sricharunrat, Vajarabhongsa Bhudhisawasdi, Mathuros Ruchirawat, Niramol Savaraj

**Affiliations:** 1 Laboratory of Environmental Toxicology, Chulabhorn Research Institute, Laksi, Bangkok 10210, Thailand; d10120101@cgi.ac.th (A.T.); Panida@cri.or.th (P.N.); varabhorn@cri.or.th (V.P.); 2 Chulabhorn Graduate Institute, Laksi, Bangkok 10210, Thailand; 3 Division of Hematology/Oncology, Miami Veterans Affairs Healthcare System, Miami, FL 33125, USA; chunjingwu@hotmail.com (C.W.); YLi4@med.miami.edu (Y.-Y.L.); Mwangpaichitre@med.miami.edu (M.W.); 4 Sylvester Comprehensive Cancer Center, University of Miami Miller School of Medicine, Miami, FL 33136, USA; 5 Department of Surgery, University of Miami Miller School of Medicine, Miami, FL 33125, USA; 6 Center of Excellence on Environmental Health, Toxicology (EHT), Ministry of Education, Bangkok 10300, Thailand; 7 Laboratory Unit of Pathology, Chulabhorn Hospital, Laksi, Bangkok 10210, Thailand; sri.thaniya@gmail.com; 8 Department of Surgery, Faculty of Medicine, Khonkaen University, Khonkaen 40000, Thailand; joevajara@gmail.com; 9 Laboratory of Chemical Carcinogenesis, Chulabhorn Research Institute, Laksi, Bangkok 10210, Thailand

**Keywords:** ASS(-)Hepatocellular carcinoma, ADI-PEG20, 5-FU, thymidylate synthase, ASS re-expression, pyrimidine metabolism

## Abstract

Argininosuccinate synthetase (ASS), a key enzyme to synthesize arginine is down regulated in many tumors including hepatocellular carcinoma (HCC). Similar to previous reports, we have found the decrease in ASS expression in poorly differentiated HCC. These ASS(-) tumors are auxotrophic for arginine. Pegylated arginine deiminase (ADI-PEG20), which degrades arginine, has shown activity in these tumors, but the antitumor effect is not robust and hence combination treatment is needed. Herein, we have elucidated the effectiveness of ADI-PEG20 combined with 5-Fluorouracil (5-FU) in ASS(-)HCC by targeting urea cycle and pyrimidine metabolism using four HCC cell lines as model. SNU398 and SNU387 express very low levels of ASS or ASS(-) while Huh-1, and HepG2 express high ASS similar to normal cells. Our results showed that the augmented cytotoxic effect of combination treatment only occurs in SNU398 and SNU387, and not in HepG2 and Huh-1 (ASS(+)) cells, and is partly due to reduced anti-apoptotic proteins X-linked inhibitor of apoptosis protein (XIAP), myeloid leukemia cell differentiation protein (Mcl-1) and B-cell lymphoma-2 (Bcl-2). Importantly, lack of ASS also influences essential enzymes in pyrimidine synthesis (carbamoyl-phosphate synthetase2, aspartate transcarbamylase and dihydrooratase (CAD) and thymidylate synthase (TS)) and malate dehydrogenase-1 (MDH-1) in TCA cycle. ADI-PEG20 treatment decreased these enzymes and made them more vulnerable to 5-FU. Transfection of ASS restored these enzymes and abolished the sensitivity to ADI-PEG20 and combination treatment. Overall, our data suggest that ASS influences multiple enzymes involved in 5-FU sensitivity. Combining ADI-PEG20 and 5-FU may be effective to treat ASS(-)hepatoma and warrants further clinical investigation.

## 1. Introduction

As reported in 2008, hepatocellular carcinoma (HCC) is the single most common cause of cancer deaths, especially in Asia [1,2]. Most recently in 2016, HCC was ranked as the third most common cancer related death in Asia-Pacific region [3]. Due to late diagnosis and poor response to systemic treatment, life expectancy is less than a year once a patient manifests symptoms [4,5,6]. However, some factors such as stage and disease could predict the survival rate after diagnosis [7]. Treatment of HCC includes chemoembolization, radiofrequency, microwave or nanoknife ablation, Y90 radioembolization and surgical resection [8,9]. These treatments are possible only if the patient has adequate liver reserve and sufficient performance status. Furthermore, these treatments are neither available nor affordable in rural areas where the majority of cases are found. Liver transplantation is another option if the patient has good performance status and meets the criteria for transplant. However, donors can be scarce. Chemotherapy remains the option for patients with large or multifocal HCC and is more applicable to patients in regions where medical facilities are limited. Sorafenib, a multi-kinase inhibitor, has been used as an effective systemic treatment for advanced HCC, but the response is limited, with prolongation of only two months beyond the median survival rate [10,11]. Unfortunately, it is generally not affordable in rural areas. These findings clearly illustrate a very large need in the treatment options for HCC.

We have previously shown that the majority of melanomas and mesotheliomas do not express argininosuccinate synthetase (ASS), a key enzyme in the urea cycle that catalyzes the synthesis of arginine from citrulline [12,13]. An arginine degrading enzyme (arginine deiminase (ADI-PEG20)) that depletes extracellular arginine can selectively kill ASS-negative tumors, while sparing ASS-expressing normal cells [14,15]. While certain HCCs show low or absent ASS expression, and must rely on exogenous arginine for survival [16], in our clinical trial, we have found that tumor cells can undergo autophagy and subsequently turn on the *ASS* gene, resulting in drug resistance. Therefore, to increase the efficacy of ADI-PEG20, a combination with other agent(s) is needed to evade autophagy and to re-direct the cells toward apoptosis [13,14,17,18].

Here, we show that the withdrawal of arginine can inhibit the growth of ASS-negative hepatocellular carcinoma (ASS(-)HCC). It is important to note that, ADI-PEG20 can down-regulate thymidylate synthase (TS) and interfere with pyrimidine synthesis. The combination of irreversible inhibition of TS (using 5-FU) with ADI-PEG20 significantly killed ASS(-)HCC. 5-FU is a well-known chemotherapeutic agent, effective against a wide variety of cancers [19]. 5-FU active metabolite fluorodeoxyuridine monophosphate (FdUMP) is known to form a stable ternary complex with TS [20,21], and hence block the access of deoxyuridine monophosphate (dUMP) to the binding site and hinder the conversion of dUMP to dTMP. The net result is depletion of dTTP and increased in dUTP, which cause an imbalance in the deoxynucleotide pool resulting in lethal DNA damage [12,20].

Here we show that the combination of ADI-PEG20 with 5-FU can induce cell apoptosis by targeting the enzymes in the urea cycle and pyrimidine metabolism in ASS(-)HCC.

## 2. Results

### 2.1. Determination of ASS Expression Level among Cancer Cell Types

**Figure 1:**
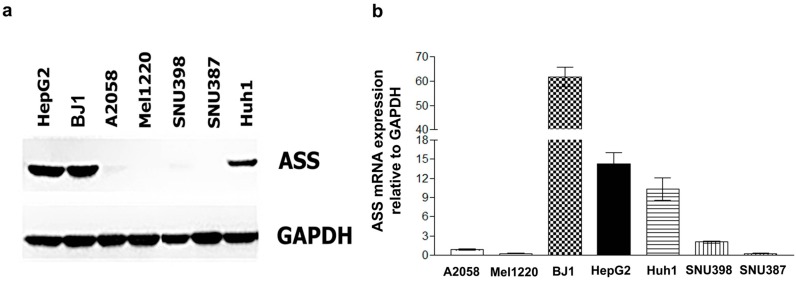
ASS expression in different cell lines compared to known ASS expression related the GAPDH expression, (ASS positive (BJ-1), moderate (A2058), and no expression (Mel1220)). (**a**) ASS protein expression; and (**b**) *ASS* mRNA expression in hepatoma cell lines SNU398 and SNU387 show low and absent ASS expression, while HepG2 and Huh-1 show high *ASS* expression.

Along with others, we have shown that the effectiveness of ADI-PEG20 depends on the levels of ASS expression [17,22,23]. Thus, we assayed ASS expression in a panel of four HCC cell lines, and compared to BJ-1 (normal immortalized fibroblast), A-2058 and Mel1220 which are known to express high, low and absent of ASS expression, respectively [15]. Our results showed that SNU387 possesses the lowest levels (absent in Western blot) followed by SNU398, while HepG2 and Huh-1 possess high ASS expression levels similar to BJ-1 (Figure 1a). In this manuscript, SNU387 and SNU398 will be defined as ASS(-). The result of Western blot also corresponded with that of qRT-PCR (Figure 1b).

### 2.2. The Levels of ASS Expression Dictate the Sensitivity to Arginine Deprivation

**Figure 2:**
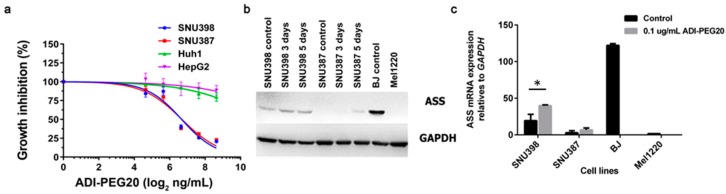

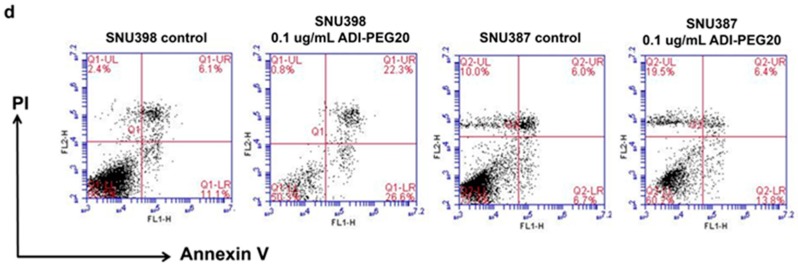
Effects of ADI-PEG20 treatment on HCC cell lines: (**a**) IC_50_ of ADI-PEG20 treatment for 72 h in ASS(-) and (+)HCC. Data presented are the mean ± SEM, from three independent experiments; (**b**) ASS inducible protein expression after 0.1 µg/mL ADI-PEG20 treatment for three and five days in SNU398 and SNU387 cell lines; (**c**) Inducible effects on ASS mRNA expression in ASS(-)HCC after 0.1 µg/mL ADI-PEG20 treatment for 72 h compared to positive control (BJ-1) and negative control (Mel1220). *ASS* mRNA expression presented are the mean ± SEM from three independent experiments with * *p <* 0.05; (**d**) Total apoptosis cell death (%) after ADI-PEG20 treatment for 72 h in SNU398 and SNU387 compared to no treatment.

We determined the growth inhibitory effect of ADI-PEG20 (which degrades arginine in the media) in four cell lines. As predicted from our previous data with other cell lines, SNU387 (ASS(-)) and SNU398 (low ASS) are sensitive to ADI-PEG20 with the inhibitory concentration (IC_50_) of 0.1 µg/mL (Figure 2a). In contrast, Huh-1 and HepG2, which express ASS, are not affected by ADI-PEG20 treatment (Figure 2a). We have previously shown that arginine in the media can influence ASS expression and can impede the growth inhibitory effect of ADI-PEG20. Here, we showed that this also occurs in SNU387 and SNU398 both at the protein and mRNA expression (Figure 2b,c). The increase was seen more in SNU398 but barely visualized in SNU387 until Day 5. The increase in mRNA was only significant (*p* < 0.05) in SNU398. Nevertheless, this effect is not reflected in the growth inhibition of melanoma [13,24], most likely because the level of ASS protein induction is too low to affect growth inhibition. Next, we determined whether the arginine deprivation results in apoptosis. Our data demonstrated that ADI-PEG20 treatment produces apoptosis in 31.7% of the SNU398 cells treated and 7.5% of the SNU387 cell treated (percentage was corrected from the baseline control) (Figure 2d).

### 2.3. The Anti-Tumor Effect of 5-FU in Combination with ADI-PEG20

**Figure 3:**
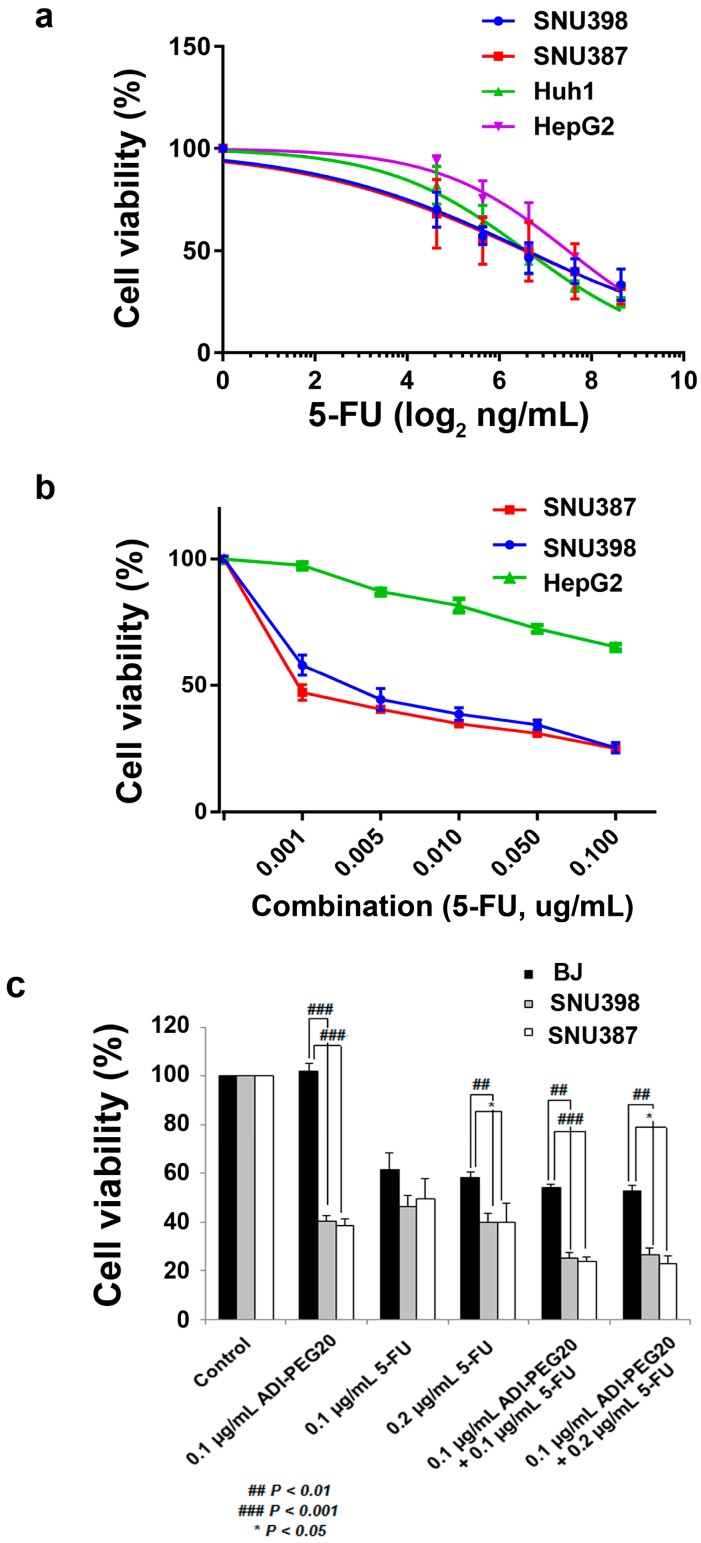
Anti-tumor effects of 5-FU and combination treatment on cell lines: (**a**) IC_50_ of 5-FU treatment in ASS(-) and (+)HCC cell lines for 72 h. Cell viability was determined as IC_50_. Data presented are the mean ± SEM from three independent experiments; (**b**) The IC_50_ of the constant 0.1 µg/mL ADI-PEG20 with various doses of 5-FU in SNU398 and SNU387; (**c**) The growth inhibiting effect of single and concurrent treatment of 0.1 µg/mL ADI-PEG20 and 5-FU treatment for 72 h in BJ-1 compared to SNU398 and SNU387. Cell viability (%) is presented as the mean ± SEM from three independent experiments.

Since treatment with ADI-PEG20 alone in SNU387 and SNU398 (Figure 2d) resulted in only 7.5–31.7% apoptotic cell death, combination treatment is needed to effectively eradicate HCC. We chose to combine ADI-PEG20 with 5-FU since this agent has been widely used alone or in combination to treat HCC, and is very well tolerated [25]. It is important to note that cells from patients with type 1 citrullinemia (ASS deficiency) show increase in pyrimidine synthesis [26]. Since 5-FU affects pyrimidine synthesis, it is likely that the antitumor effect will be improved when combined with ADI-PEG20, rather than using either agent alone. We first studied the effects of 5-FU alone (Figure 3a) and then in combination with ADI-PEG20 (Figure 3b). Our results show that the IC_50_ in all four HCC cell lines were similar, despite the difference in ASS expression (Figure 3a). SNU398, SNU387, and Huh-1 had IC_50_ at 0.1 µg/mL, while HepG2 had an IC_50_ at 0.2 µg/mL. Next, we determined whether a combination of 5-FU and ADI-PEG20 would yield an increase in cytotoxicity effect without affecting the normal cells (BJ-1). Our results show that ADI-PEG20 did not have cytotoxic effect on BJ-1 (Figure 3c). However, 5-FU at 0.1 and 0.2 µg/mL reduced the cell viability to 61% and 58% respectively and as expected, no significant changes in combination treatment *(p* = 0.22, in 0.1 µg/mL ADI-PEG20 + 0.1 µg/mL 5-FU and *p* = 0.18, in 0.1 µg/mL ADI-PEG20 + 0.2 µg/mL 5-FU). In contrast, a significant increase in growth inhibitory effect was seen when SNU398 and SNU387 were treated with 0.1 µg/mL ADI-PEG20 combined with 0.1 µg/mL 5-FU. Cell viability was reduced to 25.39% in SNU398 (*p* < 0.01) and 23.89% in SNU387 (*p* < 0.001), compared to BJ-1. The augmented cytotoxic effect persisted even with increasing 5-FU dose to 0.2 µg/mL (*p* < 0.001, for SNU 398 and *p* < 0.05, for SNU387, compared to BJ-1). It is noteworthy that cytotoxic effect of 5-FU remains the same despite increasing dosage from 0.1 to 0.2 µg/mL in BJ-1 while in SNU387 and SNU398 the cytotoxic effect increase with increasing dosage (*p* < 0.01). This is expected since both SNU387 and SNU398 divided faster than BJ-1 and hence were more sensitive to 5-FU.

### 2.4. ADI-PEG20 and 5-FU Combination Effectively Increase Cell Death

**Figure 4:**
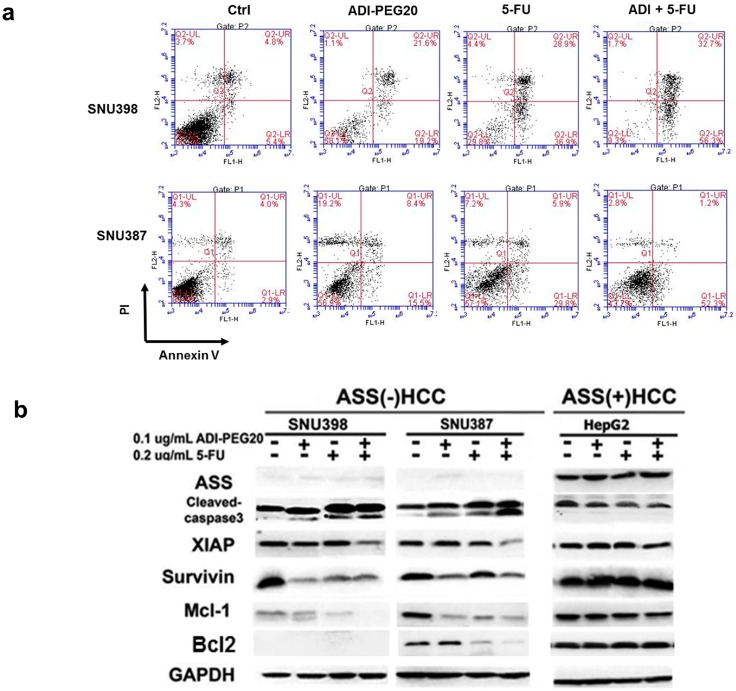

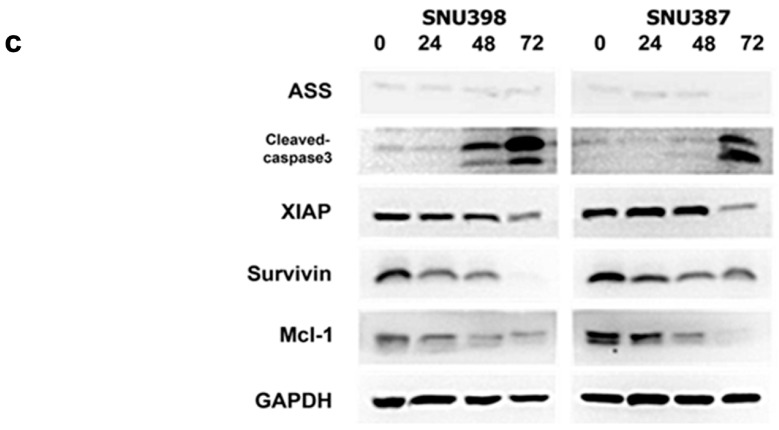
Effects of the combination treatment on cell death: (**a**) Total apoptosis (%) of the single and combined treatment (0.2 µg/mL 5-FU with 0.1 µg/mL ADI-PEG20) in SNU398 and SNU387 for 72 h determined by flow cytometry using AnnexinV/PI; (**b**) Effect of single and combination treatments for 72 h on apoptotic-related proteins, cleaved-caspase-3, XIAP, survivin, Bcl-2 and Mcl-1 in SNU398, SNU387 and HepG2; (**c**) Progression of apoptosis induction in SNU398 and SNU387 treated with the combination 0.1 µg/mL ADI-PEG20 and 0.2 µg/mL 5-FU up to 72 h.

We next determined whether the combination treatment also results in increased apoptotic cell death. Cells were treated with ADI-PEG20 (0.1 µg/mL), 5-FU alone (0.2 µg/mL) and in combination for 72 h. Apoptosis was determined by flow cytometry using AnnexinV/PI (as described in the Method Section). As shown in Figure 4a, ADI-PEG20 alone produced apoptosis in 30.6% of the SNU398 cell treated and 17% of the SNU387 cell treated, while 5-FU alone generated apoptosis in 55.6% (SNU398) and 28.8% (SNU387) of the cell treated. The combination treatment increased apoptosis to 78.8% and 50.6%, respectively. All values are adjusted with baseline control. In contrast, in BJ-1, this effect could not be seen significantly since the IC_50_ was not increased further when the combination treatment applied, compared to 5-FU treatment alone (Figure 3c). Overall, our data suggest that in HCC with low ASS expression, combination treatment improves therapeutic efficacy over treatment with either agent alone.

Since AnnexinV detects early apoptosis, we next studied whether apoptosis is also involved caspase cascade. Figure 4b shows cleaved-caspase-3 in SUN398 and SNU387. Corresponding with the AnnexinV data, cleaved-caspase-3 is highest in the combination treatment (0.1 µg/mL of ADI-PEG20 and 0.2 µg/mL of 5-FU) for both SNU398 and SNU387 cell lines, with no discernable changes in HepG2 cell line. This supports the hypothesis that the combination treatment (0.1 µg/mL ADI-PEG20 and 0.2 µg/mL 5-FU) is more effective in cell lines that express low ASS. Since we had previously shown that treatment with ADI-PEG20 in ASS(-) cells decreased anti-apoptotic proteins and increased pro-apoptotic proteins in melanoma cells, we examined whether this alteration also occurs in HCC cell lines. We examined XIAP, survivin, Bcl-2 and Mcl-1 in these cells when treated with ADI-PEG20 alone, 5-FU alone and in combination. These proteins were chosen because they were altered in our previous study [15,18,24]. As shown in Figure 4c, the level of XIAP decreases after combinations in both cell lines. Similar to melanoma cells, survivin decreased after ADI-PEG20 treatment and decreased further in the combination treatment. Mcl-1 exhibited a greater decrease after all treatments and almost no discernable after combination treatment. SNU398 did not express Bcl-2, which could explain the higher number of apoptosis upon 5-FU treatment. SNU387 expressed Bcl-2, but less so after the combination. Alteration of these anti-apoptotic proteins occurred at 48 h. (Figure 4c) and corresponded with the appearance of cleaved-caspase-3. By contrast, there were no significant changes in the anti-apoptotic proteins in HepG2 that express ASS. Overall, the decrease in anti-apoptotic proteins triggered by arginine deprivation contributes to increased apoptosis in the combination treatments.

### 2.5. Effects of ADI-PEG20 on the Key Enzymes Involved in 5-FU Sensitivity

**Figure 5:**
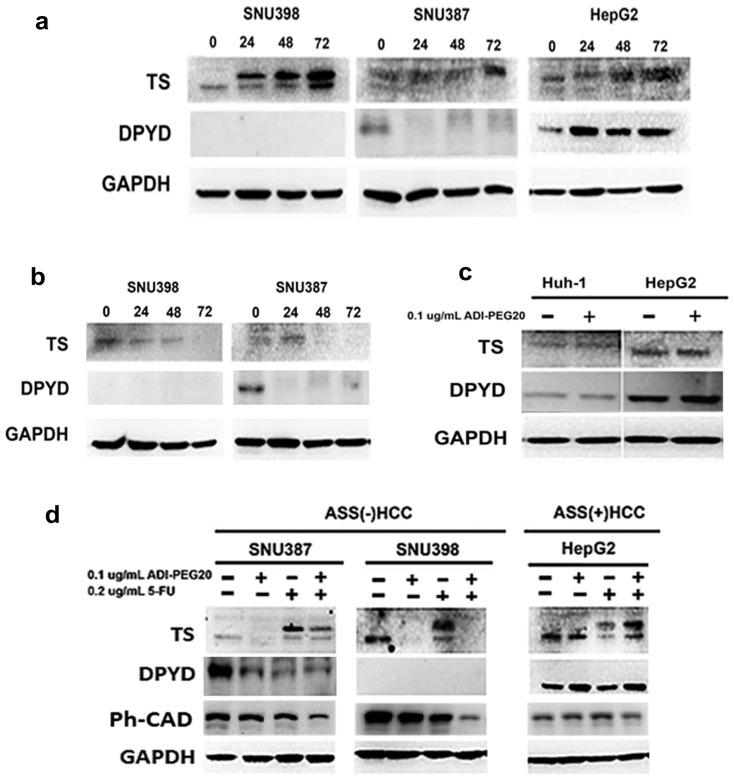
Effects of ADI-PEG20 and 5-FU on the key enzymes in pyrimidine biosynthesis: (**a**) immunoblot of TS and DPYD at 24, 48 and 72 h after treatment with 0.2 µg/mL of 5-FU in SNU398 and SNU387 compared to HepG2; (**b**) immunoblot of TS and DPYD at different time intervals in SNU398 and SNU387 after exposure to 0.1 µg/mL of ADI-PEG20; and (**c**) immunoblot of TS and DPYD after exposure to 0.1 µg/mL ADI-PEG20 in two ASS(+)HCC, Huh-1 and HepG2, for 72 h; (**d**) Effect of combination treatment with 0.1 µg/mL ADI-PEG20 and 0.2 µg/mL 5-FU on TS and DPYD in SNU398 and SNU387 for 72 h, compared to HepG2.

5-FU is known to inhibit thymidine synthase (TS), a key enzyme in pyrimidine synthesis. The expression of this enzyme has been shown to correlate with 5-FU sensitivity [20]. Another enzyme, dihydropyrimidine dehydrogenase (DPYD) which catabolizes uracil and 5-FU by reducing the 5,6 double bond of uracil, plays a key role in 5-FU bioavailability [27,28]. High levels of this enzyme in tumors correlated with resistance to 5-FU [29,30]. However, in normal tissue, DPYD deficiency results in a long terminal half-life of 5-FU and hence severe toxicity [31]. There were no correlations between the DPYD levels in normal tissue and their tumors in the same patients. Both TS and DPYD were independent factors in determining 5-FU sensitivity [32]. We examined the possible effect of arginine deprivation on the expression of TS and DPYD in tumors with ASS(-) and ASS(+) cell lines. Our results indicate that 5-FU treatment increased TS in all cell lines, regardless of the level of ASS expression. TS level began to rise at 24 h and progressively increased at 48 and 72 h (Figure 5a). In contrast, ADI-PEG20 treatment down-regulates TS levels in ASS(-) cell lines (Figure 5b). This was best seen at 48 h and continued to decrease at 72 h. As expected, ADI-PEG 20 treatment had no effect on TS levels in ASS(+) cell lines (Huh-1 and HepG2) (Figure 5c). Combination treatment resulted in further decreases in TS in ASS(-) cell lines but showed no effect in ASS(+) cell lines. This may contribute to the potentiation effect observed in the combination treatment in ASS(-) cell lines. We then examined the effect of ADI-PEG20 in DPYD. SNU398 did not express DPYD. This may explain the higher percent of cell death and increased cleaved-caspase-3 seen in SNU398 cell lines when treated with 5-FU alone. SNU387 expressed DPYD, but the levels of DPYD decreased after 5-FU and combination treatment. In HepG2, the levels of DPYD showed no discernable change (Figure 5d). Overall, down regulation of TS and DPYD in ASS(-) cell lines contributed to greater cytotoxicity in the combination treatment arm.

Arginine is generated from citrulline via ASS and aspartate to form arginiosuccinate, followed by argininosuccinate lyase to generate arginine. Thus, in ASS deficient cell lines, aspartate level is increased. Consequently, aspartate is re-routed for pyrimidine synthesis by activation of CAD (carbamoyl phosphate synthase 2, aspartate transcarbamylase and dihydroorotase) complex [12]. We determined whether alteration of this enzyme complex also seen after ADI-PEG20 treatment. Corresponding to previous published data [26], ASS(-) cell lines had a higher level of CAD enzyme expression than the HepG2 cell line. This enzyme complex progressively decreased after ADI-PEG20 treatment, with the lowest expression level seen with the combination. We conclude that shutting down the pyrimidine synthesis most likely explains the increased cell death seen with 5-FU and ADI-PEG20.

### 2.6. Reconstitution of ASS Expression Affect the Sensitivity to Combination Treatment with ADI-PEG20 Plus 5-FU Treatment as Well as the Enzyme Involved in the De Novo Pyrimidine Synthesis

**Figure 6:**
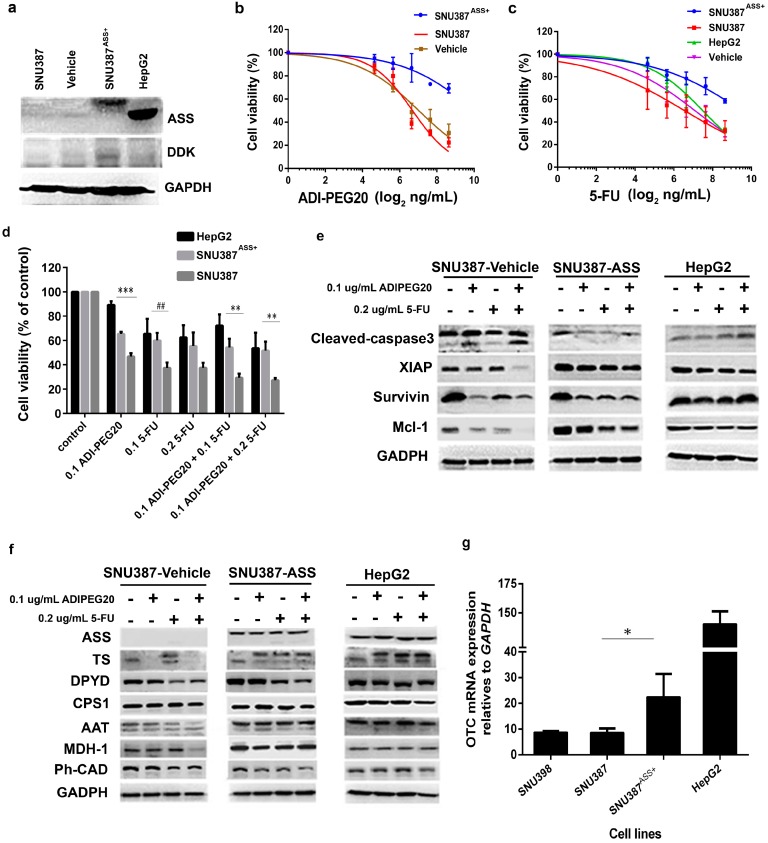
Effects of ASS expression level on the efficacy of the combination treatment and pyrimidine metabolism: (**a**) SNU387^ASS+^ after transfection with pCMV6-Entry expression vector express ASS compared to SNU387 (parental-SNU387), vehicle (empty plasmid) and positive control (HepG2); (**b**) Cell viability (%) of SNU387^ASS+^ treated with ADI-PEG20 at various doses for 72 h; (**c**) Single 5-FU treatment at various doses for 72 h. Data presented are the mean ± SEM from three independent experiments; (**d**) Cell viability (%) of SNU387^ASS+^ after single and combination treatment with ADI-PEG20 and 5-FU. Each bar graph represents the mean ± SEM from three independent experiments (*** *p* < 0.0005, *^##^ p* < 0.001, ** p < 0.005); (**e**) Effect of single and combination treatments for 72 h on apoptotic-related proteins in SNU387^ASS+^ (SNU387-ASS) compared to SNU387-vechicle and HepG2; (**f**) Effect of ASS expression level in SNU387^ASS+^ (SNU387-ASS) on pyrimidine metabolic enzymes and urea cycle; TS, DPYD, CPS1, AAT, MDH-1 and Ph-CAD expression determined in SNU387^ASS+^ by Western blot; (**g**) Inducible OTC mRNA expression in SNU387^ASS+^. Each bar graph represents the mean ± SEM from three independent experiments, (* *p* < 0.05).

To verify that ASS expression in HCC cell lines affects pyrimidine synthesis and hence 5-FU sensitivity, we transfected ASS into SNU387 using plasmid pCMV6-ASS (as stated in the Method Section); pCMV6 with no ASS was used as control. The levels of ASS expression in this transfected cell (SNU387^ASS***+***^) compared to parental SNU387 transfected with empty vector and HepG2 are shown in Figure 6a. The position of the molecular weight of ASS protein of the transfected ASS is higher due to DDK tag. The antitumor effect of 5-FU alone, ADI-PEG20 alone and in combination are shown in Figure 6b–d. As expected SNU387^ASS***+***^ is resistant to ADI-PEG20. At 0.1 µg/mL of ADI-PEG20 over 70% of cells were still viable. This ASS transfectant is also more resistant to 5-FU and resembles those from HepG2. As expected, the cytotoxic effect of combination treatment in these ASS transfected cells was not significantly different from treatment with 5-FU alone (*p* = 0.144 (0.1 µg/mL 5-FU alone and 0.1 µg/mL 5-FU in combination), *p* = 0.288 (0.2 µg/mL 5-FU alone and 0.2 µg/mL 5-FU in combination)) (Figure 6d). We next examined the anti-apoptotic proteins and cleaved-caspase-3 before and after treatment. Our results demonstrate that reconstitution of ASS abrogated the changes in two anti-apoptotic proteins (XIAP and Mcl-1) with lesser change seen on survivin (Figure 6e). It is important to note that no cleaved-caspase-3 was detected in the transfected cells. Our results confirm that ASS expression is a key factor for both ADI-PEG20 and 5-FU sensitivity.

**Figure 7:**
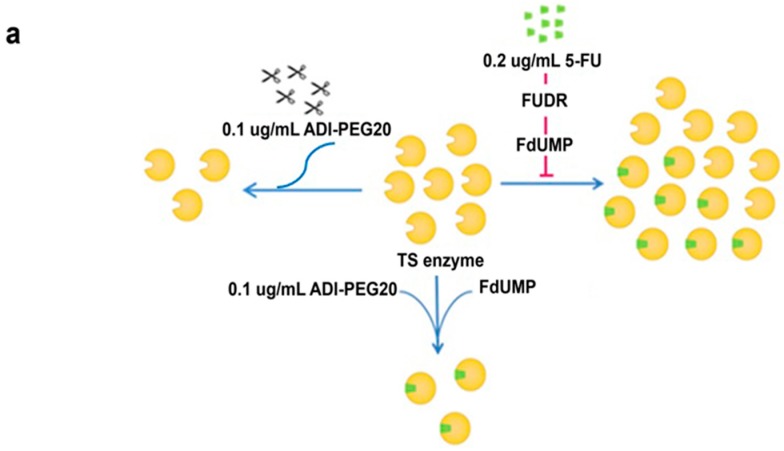

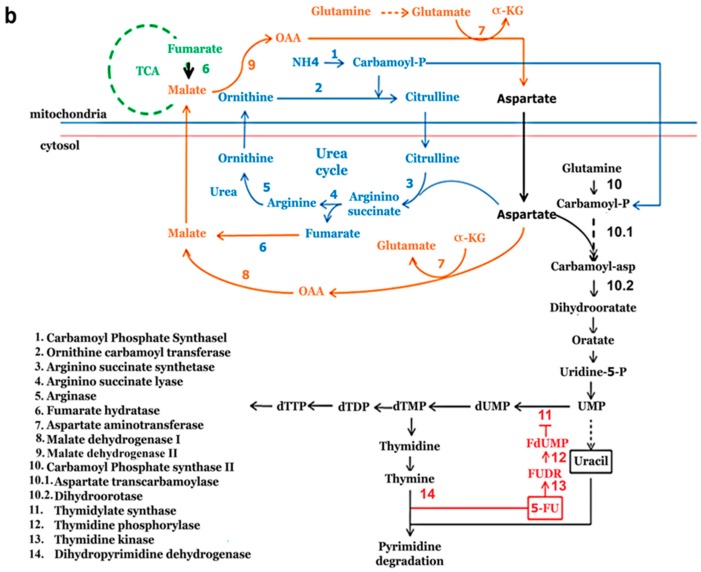
Proposed mechanism of the combined treatment on urea cycle, pyrimidine metabolism and aspartate-malate shuttle: (**a**) The mechanism of action of ADI-PEG20 reduces TS enzyme expression; and 5-FU generates active metabolite, FdUMP, to deactivate TS. Combined both treatments reduce amount of TS and make it more feasible for FdUMP to completely deactivate TS enzyme hence shut off pyrimidine synthesis; (**b**) In relationship between the urea cycle and pyrimidine metabolism, aspartate and carbamoyl phosphate distribute via both pathways. (Solid arrows define its consequences, dot arrows define some steps during the process and T bar arrow defines the inhibition).

Next, we studied whether ASS re-expression can influences the levels of TS and DPYD. Our results demonstrated that the baseline level of TS is slightly lower in the transfected cell line (Figure 6f). However, ADI-PEG20 treatment had no effect on TS. The amount of TS increased after 5-FU, and there were no changes after combination, similar to observations in HepG2. DPYD showed no changes in the transfected cells, but decreased after 5-FU and combination treatment. Overall, our data indicate that reconstitution of ASS abrogated the negative effect of ADI-PEG20 on TS but had no discernable effect on DPYD. Next, we examined other enzymes involved in the urea cycle as well as the aspartate–malate shuttle, which can influence de novo pyrimidine biosynthesis (see diagram in Figure 7). In normal cells, the first step in the urea cycle requires ammonia and carbamoyl phosphate synthase I (CPS1) to form carbamoyl phosphate. This reaction also requires the allosteric effector *N-*acetylglutamate, formed by acetyl-CoA and glutamic acid. The next enzyme, ornithine transcarbamoylase (OTC) catalyzes the reaction of ornithine with carbamoyl phosphate to form citrulline which then exits mitochondria to generate argininosuccinate via ASS and aspartate as mentioned above. Arginine is catabolized by arginase to form urea and ornithine (ORN), which can enter mitochondria to regenerate citrulline (see diagram in Figure 7). Thus, in ASS deficient cell lines, we expect that the OTC and CPS1 levels may be altered. In addition, the excess aspartate can also be converted to oxaloacetate (OAA) and to malate via aspartate aminotransferase (AAT) and malate dehydrogenase (MDH-1). Malate can then be used in the TCA cycle. We assayed these four enzymes by immunoblot. As shown in Figure 6f, the levels of *OTC* mRNA increase in the transfected cell line, but are still lower than HepG2, which intrinsically expresses ASS, while the levels of CPS1 show no change (Figure 6g). This is expected since ASS expression leads to an uninterrupted urea cycle. Thus *OTC* mRNA is increased. We did not use immunoblot to detect OTC due to lack of a suitable antibody. We speculate that the levels of CPS1 show no change because CPS1 requires allosteric effector *N-*acetylglutamine (generated from acetyl CoA and glutamic acid) to function [33]. In ASS(-) cell lines, glutamic acid may be used to compensate for lack of arginine, while carbamoyl phosphate is needed for pyrimidine synthesis. The net effect was no significant changes in CPS1 with or without ASS expression. The levels of AAT and MDH-1 showed a slight increase in the transfected cells but no significant changes after treatment. However, in parental cells, both AAT and MDH-1 decreased after combination treatment (Figure 6f). This was expected, since there is no excess aspartate after reconstituting ASS expression. Aspartate was used for both arginine synthesis and aspartate-malate shuttle, while, in the ASS(-) parental cells, excess aspartate was used for both pyrimidine synthesis and aspartate-malate shuttle.

Overall, disruption of the urea cycle due to down regulating ASS expression can result in metabolic changes that affect pyrimidine biosynthesis.

### 2.7. ASS Expression in HCC Specimens from Thai Patients

**Figure 8:**
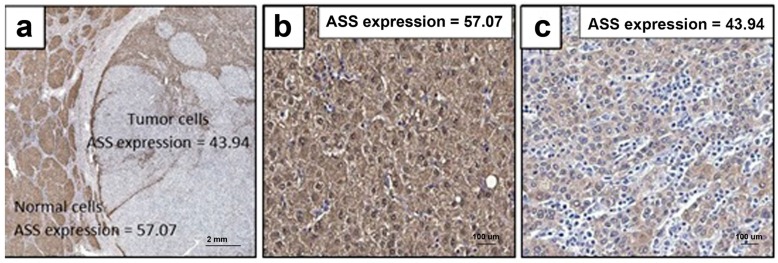
Representative immunohistochemical detection of ASS expression in paraffin-embedded sections from 40 HCC patients, intensity of ASS staining in HCC tumor cells is lower than in normal (**a**); ASS expression in normal liver (**b**); and HCC tumor cells (**c**) (100× magnification).

Since HCC show marked variation in ASS expression, it is important to know whether this wide variation also exists and is biologically relevant in human tumor samples. We assayed ASS expression in HCC from 40 Thai patients and found that the level of cytoplasmic ASS staining in the tumor was lower than in the normal liver (Figure 8). When we quantified the ASS expression level using relative fluorescence intensity, HCC showed significantly decreased ASS expression (43.94 ± 1.46, *p* < 0.05) compared to the normal liver (57.07 ± 1.47), with a mean tumor/normal ASS expression ratio of 0.79 (Table 1). To assess the ASS expression level in relation to clinical pathological feature of HCC, we stratified the ASS expression in HCC as above (high) or below (low) the mean ratio. The results are summarized in Table 2, which shows 18 (45%) of 40 patient specimens with low-ASS expression and the remaining 22 (55%) with high-ASS expression. No association was found between levels of ASS expression and factors such as gender, age, viral hepatitis status, lympho-vascular invasion, lymph node metastasis, and Tumor, node and metastasis (TNM) stage (Table 2). In HCC, however, levels of ASS expression and the degree of tumor differentiation were significantly associated (*p* = 0.032). Overall, our results are consistent with previously published data which showed that tumors with low ASS expression are more aggressive. Whether ASS expression can also be used as a marker to identify more aggressive HCC is not known and will require a large sample size.

## 3. Discussion

We have previously shown that ADI-PEG20, which degrades arginine to citrulline, has antitumor activity in melanoma [34]. The activity is seen primarily in ASS(-) tumors. Similar findings also apply to HCC on ADI-PEG20 treatment [35]. In a large randomized trial, Yang et al. found that heavily pretreated patients who had maintained undetectable arginine levels for 4 weeks were stable and had a median survival of 10 months [36]. In a recent study presented at the American Society of Clinical Oncology, ADI-PEG20 showed no significant antitumor activity compared to placebo. However, the study did not stratify ASS as positive or negative, but in the subset analysis, an antitumor effect was seen in ASS-negative patients and in patients who maintained non-detectable arginine levels. Overall, ADI-PEG20 has activity in HCC, most likely in those who have low or absent ASS expression, and maintain no detectable arginine levels in their serum.

In melanoma, we have found that, resistance to ADI-PEG20 is due to the re-expression of ASS. Whether this also occurs in HCC is not known, since no re-biopsy was performed in those patients [34]. In our study, we found an increase of ASS expression at the mRNA and protein levels in both cell lines that exhibited very low levels of ASS expression (SNU398 and SNU387) after exposure to ADI-PEG20. This may explain the low antitumor activity observed clinically [37]. Nevertheless, ADI-PEG20 shows both cytostatic and cytocidal effects in these low ASS-expressing HCC in vitro. ASS is regulated by both epigenetic and transcription factors. ASS methylation was seen in mesothelioma, bladder cancer and ovarian cancer [12]. ADI-PEG20 also has shown better antitumor activity in mesothelioma than in melanoma. In melanoma, we found that ASS is positively up-regulated by c-Myc and down-regulated by HIF-1α, and epigenetics do not play a major role in ASS regulation [38]. In HCC cell lines, due to inducible ASS seen in 4–5 days, we speculate that ASS regulation may closely resemble that in melanoma making combination treatment necessary in order to improve therapeutic efficacy [15,23].

Since ADI-PEG20 treatment has an insignificant effect on normal cells, as evident by the minimal side effects seen clinically, its use in combination with chemotherapy or immunotherapy is possible. In this report, we have chosen to study 5-FU as our combination treatment for HCC. The rationale is generated from data reported by Rabinovich et al., which show that, in Type 1 citrullinemia and in ASS(-) cell lines, aspartate is detected and is used in pyrimidine synthesis. Hence, the increased cellular proliferation [26]. Thus, by blocking pyrimidine synthesis with 5-FU, increased antitumor effect is expected in ASS(-)HCC. In addition, the mechanism of 5-FU is well established. 5-FU is known to form FdUMP, which inhibits TS in the pyrimidine synthesis pathway. It is important to note that, this drug has been used for decades with known toxicity and is affordable and available globally to treat HCC. The response rate when used as a single agent is low, but combination with other treatment is feasible due to the low toxicity profile. Our results confirm that combination treatment with ADI-PEG20 can achieve >50% cell death in SNU387 and 79% in SNU398, compared to only 29–55% with 5-FU alone. This potentiation effect is not seen in tumor cells and normal cells which express ASS. Cell death is also confirmed by cleaved-caspase-3, which is higher in the combination arm. We also have shown that reconstituted ASS in SNU387 is able to reverse the cytotoxic effect of ADI-PEG20 and in the combination treatment, as evident by 3-(4,5-dimethylthiazol-2-yl)-2,5-diphenyltetrazolium bromide assay (MTT) and the absence of cleaved-caspase-3. Interestingly, SNU398 is most sensitive to 5-FU and combination treatment, even though these cells express higher levels of ASS than SUN387. This is most likely because SNU398 expresses much less DPYD, possesses a high level of Ph-CAD, and lacks Bcl-2.

We also investigated the underlying mechanisms involved. Previously, we have shown that ADI-PEG20 or arginine deprivation decreased the anti-apoptotic protein in ASS(-) melanoma cell lines, which can enhance apoptosis upon arginine deprivation. As with melanoma cells, we have found that both survivin and Mcl-1 declined after ADI-PEG20 treatment and further decreased upon combination treatment. All the anti-apoptotic proteins, including XIAP and Bcl-2, decreased considerably upon combination treatment. In contrast, in the ASS transfected SNU387 cell line, there was no discernable change in these anti-apoptotic proteins upon combination treatment. Overall, down-regulation of these anti-apoptotic proteins by arginine deprivation contributed to the increased apoptosis seen in the combination treatment.

As reported above, ASS deficient cells exhibited an increase in pyrimidine synthesis and cell proliferation. This is secondary to increased aspartate, which results in activation of CAD to drive pyrimidine synthesis [26,39]. We have shown that Ph-CAD expression level is higher in both SUN387 and SNU398 cell lines than in HepG2 which expresses ASS. Transfection of ASS in SNU387 decreases Ph-CAD and increases *OTC* mRNA, but not to the same levels as in cells which intrinsically express ASS. Overall, reconstituting ASS expression led to the expression of urea cycle enzymes and decreased the key enzymes complex (CAD) for pyrimidine synthesis. Since aspartate can also be used in the aspartate-malate or malate-aspartate shuttle to generate OAA via transamination from glutamate using AAT, OAA can then be converted to malate via MDH-1. During this process NADH is oxidized to NAD. Malate is then imported inside mitochondria and then reverted back to OAA by mitochondrial malate dehydrogenase (MDH-2) to OAA. NAD is reduced to form NADH and an H is released. OAA is then converted back to aspartate. During this process, ATP is produced. Both AAT and MDH-1 are slightly reduced in SNU387 compared to SNU387^(ASS+)^ which is not surprising, since most of the aspartate is used in the pyrimidine synthesis. Malate can also be generated from citrate, however, upon combination treatment, these two enzymes are markedly reduced. This is most likely due to growth arrest and no need to generate ATP. In the ASS transfected cell, neither AAT nor MDH-1 showed any changes after treatment.

One unexpected finding is that arginine deprivation decreased TS, contributing to 5-FU sensitivity (Figure 7a). The decrease in TS could be because, when there is no arginine, the cells often undergo autophagy. Hence the pyrimidine synthesis is halted.

Lastly, in HCC tumor tissue, we also found that low ASS expression correlated with poorly differentiated tumors. These findings are consistent with those published by Yang et al. stating that ASS(-)HCC showed cellular dedifferentiation as they become more malignant [40]. These findings are supported by the fact that the ASS expression is reduced during tumorigenesis and cellular dedifferentiation occurs in ASS(-) cells. These cells show regression to the primitive embryonic stage of the liver during the fetal period, at which time the level of ASS is low [40,41]. However, some studies have concluded that ASS(-)HCC gives a better prognosis and survival [36,40]. This contradicted report could be due to the definition of high or low ASS expression, survival, as well as the stage at diagnosis of the patients and treatment received. Overall, the literature supports the finding that ASS(-) tumor usually has higher proliferative and more malignant. We used an arbitrary below- and above-average value which may not be perfect, but has the advantage adjacent benign tissue for comparison thus decreasing variation attributable to technical factors. Interestingly, review of the available literature on the *ASS* gene shows that the majority of studies support the notion that few if any ASS expressing tumors are highly proliferative [40,42]. It is noteworthy that ASS also has been proposed as a tumor suppressor gene [43].

Overall, our data suggest that a combination of ADI-PEG20 and 5-FU is highly effective in low-ASS expressing HCC. While there is no in vivo study, this combination should not result in overt toxicity, since both drugs have low and known toxicity profiles. Since the available FDA-approved drug, sorafenib, has a low response rate and is not very well tolerated [44], the ADI-PEG20/5-FU combination should be considered for future clinical investigation in low-ASS expressing HCC. If this combination is proven effective, it can be used worldwide, including in poorly-served areas, as it requires neither expensive equipment nor sophisticated techniques for administration.

## 4. Materials and Methods

### 4.1. Cell Lines and Culture

Two ASS(-)HCC cell lines (SNU398 and SNU387) were obtained from National Institutes of Health, Bethesda, MD, USA, and two ASS(+)HCC cell lines (HepG2 and Huh-1) and BJ-1 fibroblast were obtained from ATCC, Manassas, VA, USA. All cell lines were cultured in Minimum Essential Medium (MEM, Thermo Fisher Scientific, Waltham, MA, USA) supplied with 10% fetal bovine serum (FBS) and 1% penicillin-streptomycin. Arginine free condition is occurred by incubating MEM media with 0.1 µg/mL of ADI-PEG 20 for 48 h. We have previous shown that at this concentration of ADI-PEG20, arginine in the media is degraded to citrulline [18].

### 4.2. Reagents

ADI-PEG20 was provided by Polaris Pharmaceutical Inc., San Diego, CA, USA. 5-FU was purchased from Clinical Pharmaceutical Service (Miami, FL, USA). Antibodies against cleaved-caspase-3, XIAP, survivin, Bcl-2, Mcl-1, DPYD, Ph-CAD and secondary mouse antibody were purchased from Cell Signaling Technology (Cell Signaling Technology, Danvers, MA, USA). Anti-GADPH and anti-DDK antibodies were purchased from Origene. Anti-TS, anti-actin, anti-CPS1, anti-AAT, and anti-MDH-1 antibodies were respectively purchased from Zymed-Invitrogen (Carlsbad, CA, USA), Sigma (St. Louis, MO, USA), Abcam (Cambridge, MA, USA). The secondary antibodies against goat and rabbit IgG were from Santa Cruz Biotechnology, Dallas, TX, USA.

### 4.3. Growth Inhibitory Assay

Cell lines were plated and treated with 0.1 µg/mL ADI-PEG20 and/or 5-FU at various concentrations. The cell viability was performed by MTT assay and represented as the percentage of viable cell count in each dosage relative to that in control group, which has been regarded as 100% cell viability. The experiments were completed at least three times independently.

### 4.4. Reverse Transcription and Real Time PCR

Total RNA was isolated using Trizol reagent (Invitrogen) and quantified using Nanodrop 2000 spectrophotometer (Thermo Scientific). The obtained RNA was converted into cDNA by following the manufacture’s instruction of iScript™ cDNA synthesis kit (BioRad, Hercules, CA, USA) and using GeneAmp PCR system 2400 (Perkin Elmer, Waltham, MA, USA). Afterwards, real time PCR was performed using MyiQ (BioRad) and iQ™ SYBR^®^ Green Supermix (BioRad) and primers for ASS, OTC and GAPDH (Sigma). Primer sequences for ASS were 5′-AACTCACGCCTCCAATCC-3′ (forward) and 5′-CATAGCCTTGTTCCTTCAGC-3′ (reverse); primer sequences for OTC were 5′-AGAAATGGTCACAACTTCATGG-3′ (forward) and 5′-AGGCAAATACTCTCCTTTCTG-3′ (reverse); primer sequences for GAPDH were 5′-CTCTCTGCTCCTCCTGTTC-3′ (forward) and 5′-GGTGTCTGAGCGATGTGG-3′ (reverse).

### 4.5. Western Blotting

Briefly, whole-cell lysates were subjected to SDS-PAGE, transferred into PVDF (Millipore, Billerica, MA, USA), and immunoblotted with various antibodies overnight. The membranes were blocked with 5% non-fat milk (BioRad). After incubation with primary antibodies and horseradish peroxide (HRP)-conjugated secondary antibodies, the membrane signals were developed using Supersignal West Femto developer (Thermo Scientific) and visualized using ChemiDoc MP System (BioRad).

### 4.6. Apoptosis Assay

Apoptosis was determined using AnexinV/PI. The experiment is performed according to manufacturer’s instruction of AnnexinV-FITC Assay Kit (AbDSerotec, Hercules, CA, USA). The data were analyzed using Accuri C6 flow cytometer and CFlow plus software (BD Biosciences, Sparks, MD, USA).

### 4.7. ASS Overexpression

SNU387 cells were respectively transfected with pCMV6 empty vector (control) and pCMV6-entry vector with *ASS* gene (pCMV6-ASS) which ASS was fused with Myc-DDK (a short polypeptide designed as a tag to detect recombinant protein) at c-terminal (Origene, Rockville, MD, USA). This could make the MW of the transfected ASS protein higher with DDK tag. This vector were mixed with lipofectamine2000 (Thermo Fisher Scientific). After 24 h, transfectants were cultured in completed MEM medium supplied with G418 (500 μg/mL).

### 4.8. Immunohistochemistry

Paraffin-embed tissues including HCC tumor tissues and normal liver tissues were cut into 3 µm thickness. The tissue slides were de-paraffinized using xylene and hydrated in a decreasing gradient of ethanol before incubating them with 3% hydrogen peroxide to block endogenous peroxidase activity for 20 min. Antigen retrieval was carried out using target retrieval solution (Dako, Carpinteria, CA, USA) followed by blocking non-specific binding with biotin blocking system (Dako) for 10 min. Thereafter, the slides were incubated with anti-ASS antibody (1:100, BD Biosciences) overnight, and biotinylated anti-mouse antibody (Dako) for 15 min, and streptavidin conjugated peroxidase for another 15 min. The chromogen development is performed using DAB Substrate-Chromogen (Dako). The slides were counterstained with hematoxylin, dehydrated with ethanol and mounted with Cytoseal-60. ASS expression on tissues slides were scored by a pathologist based on intensity of ASS positive cells in more than 4 different areas using the microscope with high power objective (100×).

### 4.9. Patient and Tissue Samples

Paraffin-embedded tissues from 40 HCC patients were gathered at Chulabhorn hospital and Srinagarind hospital. The patients included 37 males and 3 females with the median age of 58 years old (range 35–78 years old). The clinical parameters, such as gender, age, viral status, histopathological differentiation, TNM staging, lymphovascular invasion, perineural invasion were included in this study. Grading system based on Edmondson-Steiner, 4-scales grading system [45]. This study was conducted under the Institutional Ethics Committee approval according to Helsinki declaration with the project Biochemical alterations in CCA: Alteration of tumor metabolism, code 013/2559, 17 August 2016 from Human Research Ethics Committee, Chulabhorn Research Institute.

### 4.10. Statistical Analysis

Data were represented as mean ± standard error of mean (SEM). Student’s *t*-test was used to compare the two sets of data. The χ-square test was used to determine the differences of the categorical variables. For the analysis, *p* < 0.05 (*) was defined as statistical significance, and *p* < 0.01 (^##^) was defined as highly statistical significance.

## 5. Conclusions

Our data suggest that concurrent treatment of ADI-PEG20 and 5-FU in HCC with low or absent ASS expression can improve the therapeutic efficacy, compared to either agent alone. ADI-PEG20 is very well tolerated with minimal adverse effect. The toxicity profile of 5-FU is well known. Hence, this combination treatment should be feasible. This regimen, if proven effective, it can be used globally in both well-served and poorly-served region.

## Figures and Tables

**Table 1:** Immunohistochemical detection of ASS expression in HCC specimens derived from Thai patient.

Cancer	Level of ASS Expression (Arbitrary Unit)		
	Normal Liver	HCC	Ratio (Tumor/Normal)
HCC	57.07 ± 1.47 ^a^	43.94 ± 1.46 *	0.79 ± 0.03
(N = 40)	56.48 (39.38 − 83.44) ^b^	43.88 (18.21 − 75.23)	0.81 (0.27 − 1.13)

The values are expressed as the mean ± SEM ^a^ and median (min-max) ^b^; * is significant difference at *p* < 0.05 as compared to normal tissue.

**Table 2:** Association between level of ASS and clinicopathological features of HCC from Thai patients.

Variables	Patients	ASS Expression Ratio ^a^		*p*-Value ^b^
		Low	High	
**All case**	40	18	22	-
**Gender**	-	-	-	0.673
Male	37	17	20	-
Female	3	1	2	-
**Age (years)**	-	-	-	0.251
<60	25	13	12	-
≥60	15	5	10	-
**Viral status (Hepatitis B or C)**	-	-	-	0.165
Yes	29	15	14	-
No	11	3	8	-
**Tumor differentiation**	-	-	-	0.032
Grade 1	7	1	6	-
Grade 2	14	4	10	-
Grade 3	14	9	5	-
Grade 4	5	4	1	-
**Lympho-vascular invasion**	-	-	-	0.533
Yes	6	2	4	-
No	34	16	18	-
**Lymph node metastasis**	-	-	-	0.386
Yes	14	5	9	-
No	26	13	13	-
**TNM stage**	-	-	-	0.072
I–II	14	9	5	-
III–IV	26	9	17	-

^a^ The mean ASS expression ratio (HCC = 0.79) is used to categorize cases into two groups, low or high ASS expression, which have an ASS expression ratio below or above the mean, respectively; ^b^ Chi-square test.

## Abbreviations

ADI-PEG20	Pegylated arginine deiminase
dUMP	Deoxyuridine monophosphate
FDUMP	Fluorodeoxyuridine monophosphase
TS	Thymidylate synthase
DPYD	Dihydropyrimidine dehydrogenase
CPS I	Carbamoyl phosphate synthase I
OTC	Ornithine transcabamoylase
AAT	Aspartate aminotransferase
MDH 1	Malate dehydrogenase 1
MDH 2	Malate dehydrogenase 2
CAD	Carbamoyl-phosphate synthase 2, aspartate trancarbamylase and dihydrooratase complex
Ph-CAD	Phosphorylated-carbamoyl-phosphate synthase 2, aspartate trancarbamylase and dihydrooratase complex
